# Preparation of a Paeonol-Containing Temperature-Sensitive *In Situ* Gel and Its Preliminary Efficacy on Allergic Rhinitis

**DOI:** 10.3390/ijms14036499

**Published:** 2013-03-22

**Authors:** Kedan Chu, Lidian Chen, Wei Xu, Huang Li, Yuqin Zhang, Weirong Xie, Jian Zheng

**Affiliations:** 1 College of Pharmacy, Fujian University of Traditional Chinese Medicine, Fuzhou 350122, China; E-Mails: chukedan@gmail.com (K.C.); xwfjtcm@sina.com (W.X.); lihuang3413@gmail.com (H.L.); zyqfj@hotmail.com (Y.Z.); xweirong3413@gmail.com (W.X.); 2 Faculty of Rehabilitation Medicine, Fujian University of Traditional Chinese Medicine, Fuzhou 350122, China; E-Mail: clidianlab@gmail.com

**Keywords:** inclusion complex of paeonol and β-cyclodextrin, *in situ* gel, allergic rhinitis, rheological studies, thermodynamic study, nasal mucosal toxicity

## Abstract

In this paper, the optimal composition of a paeonol temperature-sensitive *in situ* gel composed of poloxamer 407 (P407) was determined, and a preliminary study of its effect on allergic rhinitis was performed. The optimal composition of the paeonol temperature-sensitive *in situ* gel included 2% paeonol inclusion, 22% P407, 2% poloxamer 188 (P188) and 2% PEG6000, as assessed by thermodynamic and rheological studies. The toad palate model was employed to study the toxicity of the paeonol temperature-sensitive *in situ* gel on the nasal mucosa. The result of this experiment showed low toxicity to cilia, which allows the gel to be used for nasal administration. The Franz diffusion cell method was used to study the *in vitro* release of paeonol and suggested that the *in vitro* release was in line with the Higuchi equation. This result suggests that the paeonol could be absorbed into the body through mucous membranes and had some characteristics of a sustained effect. Finally, the guinea pig model of ovalbumin sensitized allergic rhinitis was used to evaluate the preliminary efficacy of the gel, with the paeonol temperature-sensitive *in situ* gel showing a significant effect on the guinea pig model of sensitized allergic rhinitis (AR).

Abbreviations

P407: poloxamer 407
P188: poloxamer 188
AR: allergic rhinitis
TCM: Traditional Chinese Medicine
β-CD: β-cyclodextrin
DSC: differential scanning calorimetry
LTE4: leukotriene E4
IgE: immunoglobulin E

## 1. Introduction

Allergic rhinitis (AR), also known as hyperaesthetic rhinitis, is an allergic inflammatory disease that is caused by a variety of allergens. AR is an allergic reaction that often occurs in nasal passages during the spring, autumn and winter [1]. The clinical symptoms of AR are different in different people, but the main symptoms are nasal itching, sneezing and a runny, blocked nose. The intranasal administration of drugs has long been used for the treatment of rhinitis and nasal congestion. Intranasal administration can overcome the side effects that occur in the gastrointestinal tract and the hepatic first-pass effect. Moreover, drugs are absorbed better, because of the abundant blood and lymphatic capillaries under the nasal mucosa. Because of these properties, intranasal administration can effectively improve the bioavailability of drugs. Intranasal administration has been reported to reach similar blood concentrations as intravenous administration [2]. Most of the commercially available nasal preparations are now sprays. The scavenging effect of nasal cilia leads to a very short drug residence time on the human nasal mucosal surface (only 15–30 min) [3], which affects the clinical efficacy to some extent. Therefore, a high research value and various application prospects surround the development of clinically efficacious and safe nasal preparations.

**Figure 1:**
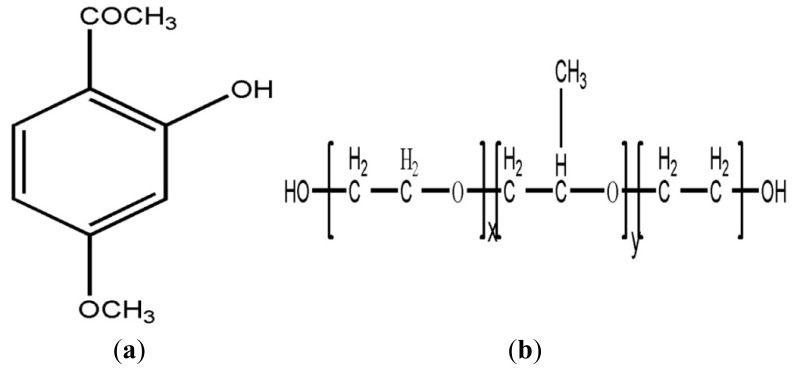
The chemical structure. (**a**) paeonol; (**b**) the triblock copolymer (polyethylene oxide (PEO)-polypropylene oxide (PPO)-PEO) block copolymer.

An *in situ* gel is made of polymer materials that have a solution or semisolid state that responds to external stimuli at the administration site. These gels also have conformations that can undergo reversible conversion to form a semisolid or solid preparation [4]. *In situ* gel types include thermo-sensitive, ion-activated and electric-sensitive, magnetic field-sensitive, ultrasonic-sensitive and chemical material-sensitive varieties. The most extensive and mature type of *in situ* gel currently used in research is the gel that reacts to changes in temperature, called a “temperature-sensitive *in situ* gel”. A temperature-sensitive *in situ* gel is liquid or semisolid at room temperature and congeals into gel as the temperature increases from room temperature to body temperature, which results in good adhesion and slow release effects [5,6]. The gel has a three-dimensional network structure that is hydrophilic and has the advantages of good biocompatibility, simple preparation, easy use, good mucosal tissue affinity and long residence time. Poloxamer 407 (P407) is one of the most widely used and most thoroughly studied polymer materials in the temperature-sensitive *in situ* gel family [7]. The structure of P407 is shown in Figure 1b; it is a non-ionic triblock copolymer (PEO-PPO-PEO) that consists of approximately 70% polyethylene oxide (PEO) and 30% polypropylene oxide (PPO), and it has an average molecular weight of approximately 11,500. P407 is soluble in water and has surface activity and a good safety level. Moreover, a certain concentration of P407 has a reverse thermal gelling property that causes it to be a liquid at low temperature and a gel when the temperature rises. Therefore, temperature-sensitive *in situ* gels that use P407 as a main component were used in this paper.

In 2001, the World Health Organization recommended glucocorticoids as the preferred drug for treatment for AR. Although the safety of nasal corticosteroid treatment has been widely recognized by the industry, its systemic side effects are still the main obstacle to its wider applications in the clinic. It has been reported that Traditional Chinese Medicine (TCM) has great advantages in adjusting immune function, and people are now giving TCM more and more attention for its positive and long-lasting effects on AR and its limited number of side effects.

Paeonol (as shown in Figure 1a) is the main active component of the root bark of *Paeonia suffruticosa* Ardr. and *Paeonia lactiflora* Pall. and the root of the entire *Cynanchum paniculatum* (Bunge) Kitag. plant. Paeonol is a low molecular weight phenolic compound that is bitter and acrid in taste. It is volatile, has poor solubility in water and has various analgesic, antioxidant and anti-inflammatory pharmacological effects [8]. The main paeonol preparations currently for sale are paeonol injections, paeonol tablets and paeonol ointments. These formulations are used for the clinical treatment of eczema, cardiovascular diseases [9], allergic rhinitis [10,11], gynecological diseases and other ailments [12].

In this study, paeonol was included by β-cyclodextrin (β-CD), and an optimum formulation of a paeonol temperature-sensitive *in situ* gel that was primarily composed of poloxamer 407 was sieved out through thermodynamic and rheological studies at the same time. We not only studied the *in vitro* release and nasal ciliary toxicity of the gel, but also examined the therapeutic effect that the nasal delivery of the paeonol-β-CD temperature-sensitive *in situ* gel has on AR. The objective of this paper was to provide a new TCM preparation to effectively treat AR.

## 2. Results and Discussion

### 2.1. The Inclusion Complex of Paeonol and β-CD

Because paeonol is volatile and has poor water solubility, the formation of complexes with β-CD protects the paeonol by cutting off contact with the surrounding solvent, which could effectively prevent oxidation and volatilizing, thus increasing its stability. Two terminals of paeonol and β-CD inclusion complex were water soluble, and the capsule interior was lyophobic—properties that could increase the water solubility, the stability and bioavailability.

**Figure 2:**
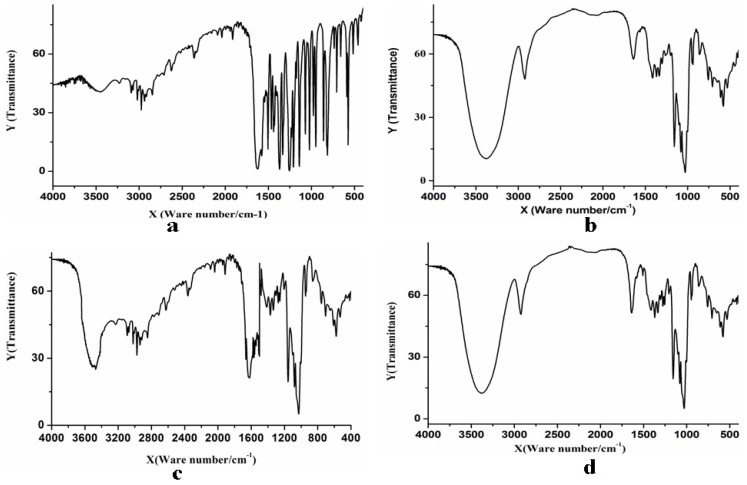
The infrared spectrograms of (**a**) paeonol, (**b**) β-cyclodextrin (β-CD); (**c**) the physico-compounds of paeonol and β-CD; (**d**) inclusion complex of paeonol and β-CD.

The infrared spectrograms of paeonol, β-CD, the physico-compounds of paeonol and β-CD and the inclusion complex of paeonol and β-CD are shown in Figure 2. Figure 2a shows that paeonol had an absorption in the range of 1504.6–1627.6 cm^−1^ and 1911.9–2099.4 cm^−1^, due to the carbonyl conjugate system in the molecular structure of paeonol. β-CD (Figure 2b) had strong absorption at 3200–3500 cm^−1^ and 800–1200 cm^−1^. The basic diffraction peaks of the physico-compounds of paeonol and β-CD (Figure 2c) had some features in common with the diffraction peaks of paeonol and β-CD. The physico-compound absorbed in the range of 3300–3400 cm^−1^, 1504.6–1627.6 cm^−1^ and 800–1200 cm^−1^ and had melting point peaks for both paeonol and β-CD. This indicated that the paeonol and β-CD were simply mixed and not interconnected. However, the inclusion complex of paeonol and β-CD (Figure 2d) was different. Some of the diffraction peaks of the inclusion complex of paeonol and β-CD disappeared, due to the screening effect that paeonol has on β-CD. However, there was still a peak characteristic of paeonol at 1627.6 cm^−1^. This may be because there was a small amount of residual paeonol in the solution, as the above results fully prove that paeonol was in the cavity of β-CD and the inclusion complex had already constituted a new object.

**Figure 3:**
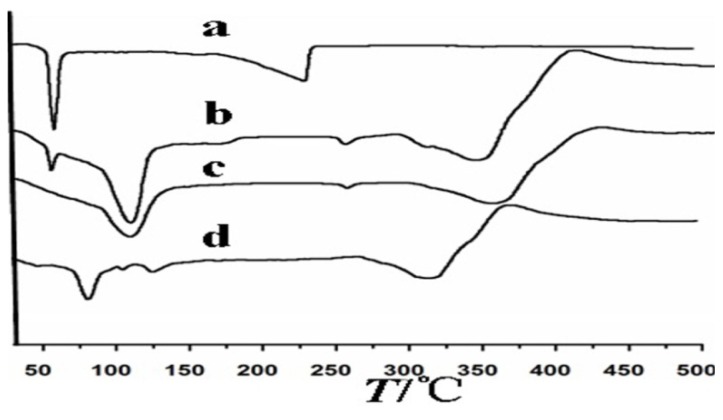
The differential thermal curves of the different samples. (**a**) paeonol; (**b**) the physico-compounds of paeonol and β-CD; (**c**) β-CD; and (**d**) the inclusion complex of paeonol and β-CD.

Figure 3 shows the differential thermal curves of paeonol (a), the physico-compounds of paeonol and β-CD (b), β-CD (c) and the inclusion complex of paeonol and β-CD (d). Paeonol had strong absorption peaks approximately 50 °C, which corresponded to its melting point peaks. The melting point peaks of β-CD appear at approximately 100 °C and 300 °C, and the physico-compounds of paeonol and β-CD had peaks characteristic of both paeonol and β-CD, possibly indicating that the two were only mixed and not interconnected. The differential thermal curves for the inclusion complex of paeonol and β-CD showed that the strong absorption peaks at approximately 50 °C had disappeared and that there was a new peak at approximately 90 °C. It could be inferred that the inclusion complex of paeonol and β-CD was different from the physico-compounds of paeonol and β-CD, because an inclusion complex of paeonol and β-CD had formed.

### 2.2. Composition Screening of the Paeonol Temperature-Sensitive *In Situ* Gel

P407 is a heat-shrink non-ionic surfactant, and its gelling mechanism is considered to be caused by tangles and stacks among the micelles when the temperature increases. A pure P407 solution can generally form a gel at temperatures below 30 °C. This is not the ideal gelling temperature, but the appropriate gelling temperature can be obtained by adjusting the ratio of PPO block and PEO block. Combining P407 with other auxiliary materials, such as poloxamer homologues (like poloxamer 188) and polyethylene glycol, can improve gel properties. For example, polyethylene glycol can increase the gelling temperature and reduce the amount of poloxamer required [13]. Therefore, P407 served as the substrate and P188 and PEG6000 served as temperature regulators for the preparation of the paeonol temperature-sensitive *in situ* gel. *T*_1_, *T*_2_ and η were chosen as indices for screening the composition.

#### 2.2.1. Influence of P407 on *T*_1_, *T*_2_ and η

**Figure 4:**
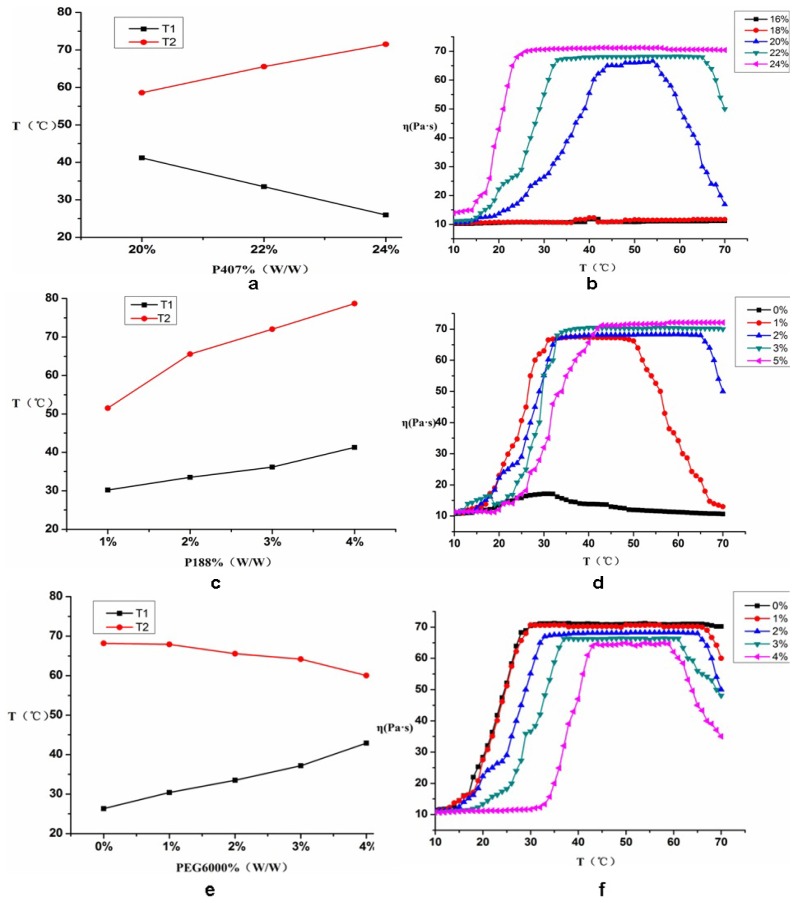
The effects of the concentration of poloxamer 407 (P407), poloxamer 188 (P188) and PEG6000 on *T*_1_, *T*_2_ and η. (**a**) the effects of different concentrations of P407 on *T*_1_ and *T*_2_; (**b**) the effects of different concentrations of P407 on η; (**c**) the effects of different concentrations of P188 on *T*_1_ and *T*_2_; (**d**) the effects of different concentrations of P188 on of η; (**e**) the effects of the different concentrations of PEG6000 on *T*_1_ and *T*_2_; (**f**) the effects of the different concentrations of PEG6000 on η.

The gelling temperature of P407 had a strong concentration dependence and a high concentration of P407 (18%–25%) was usually used to ensure sensitivity to temperature changes. The proper amounts of paeonol inclusion, 4% P188 and 2% PEG6000 were put into the solution and P407 was added to prepare a 16%, 18%, 20%, 22% or 24% solution (*w*/*w*). The results for *T*_1_, *T*_2_ and η are shown in Figure 4a,b.

Figure 4 shows that when the concentrations of P188 (4%) and PEG6000 (2%) remained constant and the concentration of P407 was 16% or 18%, the solution was still liquid despite an increase in temperature. The gelling temperature did not significantly change, which meant that it was not a gel. This may be related to the concentration of P407, which was too low to make the micelles tangle and stack with each other.

When the concentration of P407 was 20%, 22% or 24%, the gelling temperature showed a dependence on the P407 concentration: *T*_1_ decreased, *T*_2_ increased and the gel process grew wider as the P407 concentration increased. When the concentration of P407 was 24%, η did not change significantly after the temperature increased to the gelling temperature, *T*_1_. When the concentration of P407 was 22%, the gel process was wider than that of 20% P407 and the number of micelles and the chance of contact and entanglement increased, favoring the formation of the gel. Therefore, 22% P407 was selected for further study.

#### 2.2.2. The Effect of P188 on *T*_1_, *T*_2_ and η

A certain amount of paeonol inclusion, 22% P407 and 2% PEG6000 were put into the solution. The effects of 0%, 1%, 2%, 3% or 5% (*w*/*w*) P188 on *T*_1_, *T*_2_ and η were investigated. The results are shown in Figure 4c,d.

Figure 4 shows that when the P407 PEG6000 concentrations were kept constant (22% and 2%, respectively) and the P188 concentration was 0%, the sample could form a basic gel. The viscosity of this sample was low, and the gelling temperature had no significant transition point. The state tended to be a solution and was not stable. When the P188 concentrations were 2%, 6%, 10% or 14%, *T*_1_ and *T*_2_ showed an upward trend as the concentration of P188 increased, the gel process became wider and the gel became much more stable. This may be related to the increasing ability of the P407 and P188 copolymer to form micelles that tangle and stack easily with each other. When the P188 concentration reached 6%, 10% or 14%, *T*_2_ was high, the gel had a higher melting transition point and η was also higher. However, when the P188 concentration was 2%, a good gel could be formed at 33.5 °C (the temperature of the nasal cavity is 33 °C–34 °C) and the gel could melt at approximately 55 °C. This may be related to the fact that P188 might be able to change the ratio of PEO and PPO in P407. Therefore, 2% P188 was selected for further study.

#### 2.2.3. The Effect of PEG6000 on *T*_1_, *T*_2_ and η

A certain amount of paeonol inclusion, 22% P407 and 2% P188 were put into the solution. *T*_1_, *T*_2_ and η were measured when the polyethylene glycol (PEG) concentration was 0%, 1%, 2%, 3% or 4% (*w*/*w*). Figure 4e,f show the results of this study.

When the concentrations of P407 and P188 stayed the same (22% and 2%, respectively) and the concentration of PEG6000 was 0%, the sample solution formed a gel at 31.3 °C, which was lower than the optimal gel formation temperature of 33.5 °C. When the PEG6000 concentration was 1%, 2%, 3% or 4%, *T*_1_ increased, *T*_2_ decreased, the gel process gradually became narrower and η decreased as the concentration of PEG6000 increased. When the concentration of PEG6000 was 2%, *T*_1_ remained at the optimum temperature of 33.5 ± 0.5 °C, η remained at approximately 70 Pa·s and the gel was relatively stable. These results might be because the PEG molecules formed micelles with P407 after PEG6000 was added into the solution in which P407 was obviously soluble. Meanwhile, PEG significantly reduced the viscosity of the gel. Therefore, the best choice for PEG6000 concentration was 2%.

In summary, the optimal makeup of the paeonol temperature-sensitive *in situ* gel was as follows: 2% paeonol, 22% P407, 2% P188 and 2% PEG6000. Under the optimal makeup, the state of the temperature-sensitive *in situ* gel was good and was stable in three markers of validation, as *T*_1_, *T*_2_ and η were 33.5 ± 0.29 °C, 55 ± 0.56 °C, 70 ± 0.67 Pa·s, respectively.

### 2.3. *In Vitro* Release Study

The cumulative permeation of paeonol from the paeonol thermosensitive *in situ* gel was calculated according to the following Equation [14]:

(1) $$Q=2C_{0}\;{\left(D_{\text{t}}/\pi \right)}^{1/2}$$

In the formula, *Q* is the cumulative release of paeonol from the thermosensitive *in situ* gel per unit area, *C*_0_ is the initial concentration, *D* is the diffusion coefficient and *t* is the time.

**Figure 5:**
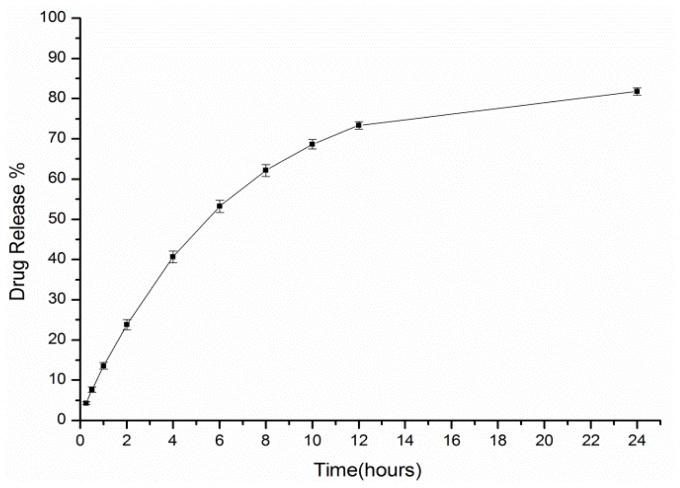
The cumulative *in vitro* release of paeonol from the temperature-sensitive *in situ* gel into a saline receiving solution.

The cumulative *in vitro* paeonol release is shown in Figure 5, which shows that the cumulative *in vitro* paeonol release gradually increased as time increased. The drug release was fast during the first 12 h and relatively stable during the remaining 12 h. The level of paeonol released within 12 h was in line with the Higuchi equation (*Q* = 147.5*t*^1/2^ − 58.01, *r* = 0.9990) by curve fitting. The curve showed that the cumulative release of paeonol was proportional to *t*^1/2^ within 12 h and was approximately proportional to *t* during the remaining time. It could be said that the inclusion complex of paeonol and β-CD could be absorbed into body through the mucous membranes and have some sustained effect.

### 2.4. Nasal Ciliary Toxicity Study of the Paeonol Temperature-Sensitive *In Situ* Gel

**Figure 6:**
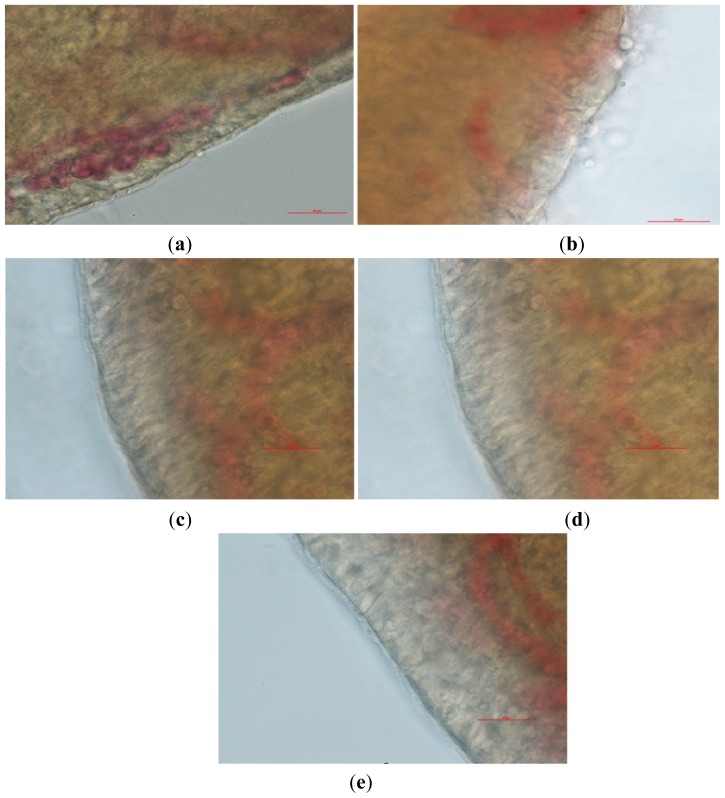
Observations of the cilia on the nasal mucosa of the toad palate. The photographs were taken at a magnification of 400×. (**a**) Physiological saline group; (**b**) 1% sodium deoxycholate group; (**c**) Naristillae Furacilini et Ephedrini group; (**d**) Paeonol temperature-sensitive *in situ* gel group; (**e**) Blank temperature-sensitive *in situ* gel group. Scale: 50 μm

Nasal cilia were observed under the microscope and the results shown in Figure 6. The cilia of the physiological saline group (Group A) and ephedrine hydrochloride group were clear, complete and lined up in order and moved actively and regularly swayed in the same direction (shown in Figure 6a,c). However, the cilia in the above groups stopped swinging, and most of their edge broke away from the nasal mucosa after treatment with sodium deoxycholate (shown in Figure 6b). It has been suggested that sodium deoxycholate has a serious destructive effect on mucosal tissues and a serious, irreversible inhibitory effect on ciliary movement. The cilia of the paeonol administration groups and the blank temperature sensitive gel group were clear, and their movement was active (shown in Figure 6d,e). This study demonstrated that the drugs and auxiliary materials had little toxicity on nasal cilia and that they could be used in a nasal delivery system. The length of continuous cilia swinging and the relative percentages of time are shown in Table 1.

### 2.5. Studies of the Paeonol Temperature-Sensitive *In Situ* Gel for the Treatment of Allergic Rhinitis

#### 2.5.1. IgE and LTE4 in the Serum

The results of the leukotriene E4 (LTE4) and immunoglobulin E (IgE) serum assays are shown in Table 2. The IgE and LTE4 levels in the sera of sensitized guinea pigs (group B to group F) were significantly higher than the IgE and LTE4 levels in the sera of non-sensitized guinea pigs (group A) (*p* <0.05). Therefore, it could be deduced that our model of allergic rhinitis was accurate. Because the IgE and LTE4 levels in the sera of the paeonol temperature-sensitive *in situ* gel group were significantly lower than those of the model group, it was suggested that the paeonol temperature-sensitive *in situ* gel may suppress allergic reactions by reducing IgE and LTE4 levels.

#### 2.5.2. Histological Examination of Nasal Mucosa

**Figure 7:**
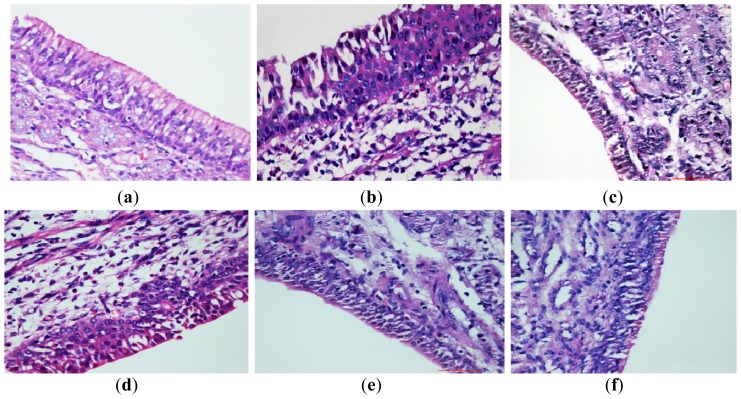
Pathological changes in guinea pig nasal mucosal tissues. (**a**) blank control group; (**b**) AR model group; (**c**) Rhinocort nasal spray group; (**d**) low-dose paeonol-β-CD temperature-sensitive *in situ* gel group; (**e**) middle-dose paeonol temperature-sensitive *in situ* gel group; (**f**) high-dose paeonol temperature-sensitive *in situ* gel group. Scale: 50 μm

An examination of the guinea pig nasal mucosal sections indicated that the nasal mucosal structures of the blank control group were intact and there was no oedema, vascular congestion or inflammatory cell infiltration in the submucosa (as shown in Figure 7a). The model group showed obvious oedema, a loose organizational structure, vascular congestion, inflammatory cell infiltration and large numbers of eosinophils (as shown in Figure 7b). The oedema, vascular congestion and eosinophil infiltration into the nasal mucosa were significantly reduced in the paeonol temperature-sensitive *in situ* gel groups and the positive control group (as shown in Figure 7d–f).

The eosinophil counts for the nasal mucosa of each group are shown in Table 3. The number of eosinophils increased significantly after allergen priming in Group B, Group C, Group D, Group E and Group F. The number of eosinophils in the medication groups was significantly reduced in comparison to the model group. These results are similar to the results of the IgE and LTE4 assays, suggesting that the paeonol temperature-sensitive *in situ* gel may treat RA by reducing inflammatory cell infiltration in the local nasal mucosa.

## 3. Experimental Section

### 3.1. Materials

Poloxamer 407 and poloxamer 188 were purchased from BASF Corporation, Germany. The PEG6000 and β-CD were purchased from Sinopharm Group Co., Ltd. (Shanghai, China). The sodium deoxycholate was purchased from Solarbio Technology Co., Ltd. (Beijing, China). The ovalbumin (egg, grade V) was purchased from Sigma-Aldrich Co. LLC. (Saint Louis, MO, USA). Methanol was purchased from Merck Company (Frankfurter Str., Darmstadt, Germany). Double-distilled deionized water was used in this paper.

A dialysis bag (MWCO 6000) was bought from USA Viskase Corporation, the CDR-34P differential scanning calorimeter was from Shanghai Precision Scientific Instrument Co., Ltd. (Shanghai, China). The FTIR 8400-S type of Fourier transform infrared spectrometer was purchased from Shimadzu, Japan. The NDJ-8S-type rotating viscometer was purchased from Shanghai Precision Scientific Instrument Co., Ltd. The XS105 electronic analytical balance was from METTLER TOLEDO. The RYJ-6A transdermal drug diffusion test instrument was bought from Shanghai Yellow Sea Drug Testing Instrument Co., Ltd. (Shanghai, China). The Nikon DS-Ri1 biological digital microscope was from Nikon Instrument Co., Ltd. (Tokyo, Japan).

### 3.2. Preparation of an Inclusion Complex of Paeonol and β-CD

In this study, a saturated aqueous solution method was employed to prepare the inclusion complex of paeonol and β-CD [15,16]. An adequate amount of paeonol was accurately weighed and sewn up together into β-cyclodextrin at a proportion of 1:8 at 45 °C for 1 h and was then refrigerated for 24 h at 4 °C to crystallize. The filtrate and the inclusion were obtained from this preparation.

### 3.3. The Characterization of the Paeonol-β-CD Complex

#### 3.3.1. Infrared Spectroscopic Analysis

Infrared spectroscopic analysis of the paeonol-β-CD complex was done, as per the literature [17]. Potassium bromide was used for tabletting. The medical grade paeonol, β-CD, the physico-compounds of paeonol and β-CD and the inclusion complex of paeonol and β-CD were analyzed by infrared spectrometry.

#### 3.3.2. Differential Scanning Calorimetry (DSC) Analysis

Differential scanning calorimetry (DSC) analysis of the paeonol-β-CD complex was done, as per the literature [18]. Five milligrams of paeonol, β-CD, the physico-compounds of paeonol and β-CD and the inclusion complex of paeonol and β-CD were weighed precisely and placed into the crucible of the DSC analyzer. Then, they were determined at 10 °C/min of the heating rate in air. α-A1_2_O_3_ was used as a reference. The differential thermal curves for each of the samples were compared.

### 3.4. Preparation of the Paeonol Temperature-Sensitive *In Situ* Gel

An appropriate cold process method [19] was employed to prepare the inclusion complex of paeonol and β-CD in the *in situ* gel. Prescribed dose of paeonol inclusion, P188 and PEG6000 were added to deionized water, stirred, dissolved and then mixed with P407. The solution was stored overnight in a refrigerator at 4 °C until the P407 was completely dissolved and the paeonol *in situ* gel was obtained.

### 3.5. Evaluation of the Paeonol Temperature-Sensitive *In Situ* Gel

#### 3.5.1. Thermodynamic Study (Gelling Temperature *T*_1_ and Gel Melting Temperature *T*_2_)

In this study, *T*_1_ and *T*_2_ were determined by a magnetic stirrer with 85-2 type (Changzhou, China) [20–22]. Ten grams of temperature-sensitive *in situ* gel solution and a stir bar were added to a vial. A micro thermometer with an accuracy of 0.1 °C was also inserted into the vial with the mercury bulb immersed in the gel solution. Its correction coefficient was 1.006. The vial was then put into a water bath set at 10 °C, and the rotation speed was set at 300 r/min. The temperature raising speed was maintained between 1 °C/min and 2 °C/min. Afterwards, the temperature at which the magnetic stir bar completely stopped rotating was defined as the gelling temperature (*T*_1_). The temperature at which the magnetic stir bar could start rotating with continuing heating was defined as the gel melting temperature (*T*_2_). The preparation was a semisolid gel from *T*_1_ to *T*_2_. This temperature range was defined as the gel process. The thermodynamic variation of the paeonol thermosensitive gel was evaluated by the determination of *T*_1_ and *T*_2_. All of the samples were measured three times in parallel, with the results expressed as the mean of the three readings.

#### 3.5.2. Rheological Properties Study

The viscosity of the paeonol thermosensitive *in situ* gel was measured by a rotational viscometer (NDJ-8S, Shanghai, China) [23]. One hundred milliliters of thermosensitive *in situ* gel that was stored at 4 °C was put into a beaker and the gel was heated to 40 °C by a controllable water bath (HH-S, Shanghai, China). The viscosity was measured every 1 °C. All of the studies were performed three times in parallel.

### 3.6. *In Vitro* Release Study

The Franz diffusion cell method [24] (RYJ-6A, Shanghai, China) was used to determine the release of the paeonol thermosensitive *in situ* gel *in vitro*. The semi-permeable membrane (molecular weight cut-off 6000, USA Viskase Corporation, Illinois, USA) was placed between the diffusion cell and the receiving cell. Then, 0.5 g of gel was placed into the diffusion cell and evenly distributed on the semi-permeable membrane surface with an area of 0.75 cm^2^. The membrane was then placed in an oven at 34 °C for 10 min and a gel was achieved. The diffusion cell was then placed in a 34 ± 0.1 °C water bath, the receiving cell was filled with warm saline solution and a speed of 300 rpm was maintained with a stir bar. All of the receiving fluid was removed, and the same amount of new receiving fluid was quickly added at 0.25, 0.5, 1, 2, 4, 6, 8, 10, 12 and 24 h. The receiving fluid was filtered through a 0.45 μm microporous membrane, and the first part of the filtrate was discarded. High-performance liquid chromatography (HPLC) [23] was used to determinate the peak area of paeonol and to calculate the paeonol content. These experiments were performed three times in parallel.

### 3.7. Nasal Ciliary Toxicity Study

The *in situ* toad palate model was used to study the nasal ciliary toxicity of the paeonol temperature-sensitive *in situ* gel [25,26]. In this experiment, 0.5 mL paeonol thermosensitive *in situ* gel was dripped onto the palate mucosa after exposing the toad palate. The drug was washed off by physiological saline after contacting the palate for 30 min. The palate mucosa were then separated, washed and placed under an optical microscope to watch the movement of the cilia. Ephedrine hydrochloride, which is generally acknowledged to be safe for cilia, was set as the negative control. Sodium deoxycholate, which is generally acknowledged as having severe ciliotoxicity, was chosen as the positive control drug. Physiological saline was chosen as the blank control.

### 3.8. Preliminary Pharmacodynamic Study

#### 3.8.1. Animals

Forty-eight guinea pigs (specific pathogen free (SPF), Hartley guinea, half male and half female, 300–400 g) were maintained under specific pathogen free (SPF) conditions. The temperature was controlled to 25 °C ± 0.2 °C, and the relative humidity was controlled at 55% ± 5%. We performed these experiments in the Animal Centre of the Fujian University of Traditional Chinese Medicine.

#### 3.8.2. Grouping and Modeling

All of the guinea pigs were acclimated for 5 days before being divided randomly into group A, B, C, D, E or F (*n* = 8). Group A was used as the physiological saline group (blank control group), group B was used as the model group, group C was used as the Rhinocort nasal spray group, group D was used as the low-dose paeonol temperature-sensitive *in situ* gel group, group E was used as the middle-dose paeonol temperature-sensitive *in situ* gel group and group F was used as the high-dose paeonol temperature-sensitive *in situ* gel group. Every guinea pig, except those in the blank control group, was intraperitoneally injected with modeling suspension (which was made up of 0.5 mg of ovalbumin (OVA) as the antigen, 30 mg of aluminum hydroxide powder as the adjuvant and 1 mL of physiological saline) once a day for 7 days. Fifty microliters of 2% egg albumen was then placed in their nasal cavities once a day for five days to elicit a reaction. The guinea pigs in the blank control group were given the equivalent volume of physiological saline in the same way.

#### 3.8.3. Administration and Sample Collection

Administration began on the second day of the seven sensitizing injections. Fifty microliters of physic liquor was dripped into the guinea pigs’ nasal cavities three times daily and was given for 11 continuous days. The guinea pigs in the blank control group were given an equivalent volume of physiological saline in the same way. After eleven days, all of the guinea pigs were fasted overnight. The guinea pigs were then anaesthetized with ketamine-diazepam by intraperitoneal injection, and the blood was aseptically obtained from the abdominal aorta. The blood-containing tubes were allowed to stand at room temperature for 2 h, and the sera were obtained by centrifugation at 3000 ×*g* for 20 min at 4 °C and stored at −80 °C. After blood collection, the turbinate and septal mucosa were obtained and fixed in 10% formalin.

#### 3.8.4. Determination of IgE and LTE4 in Serum

The serum levels of IgE and LTE4 were measured using enzyme-linked immunosorbent assay (ELISA) kits (Xitang, Shanghai, China), according to the manufacturer’s instructions. The wells were coated with 100 μL capture antibody diluted in coating buffer. The plate was sealed and incubated overnight at 4 °C. After three washes, the wells were blocked with 200 μL assay diluent at room temperature for 1 h, followed by other washes. Then, 100 μL diluted IgE and LTE4 standard and test samples were added and incubated for 2 h at room temperature. After repeated washes, the substrate was added and incubated for 20 min at room temperature, and the absorbance was measured at 450 nm using an ELISA reader (BioTek, Model ELX800, Chicagoland, IL, USA).

#### 3.8.5. Histopathological Examination of the Nasal Mucosa

The nasal mucosa was taken out from the 10% formalin. Then was paraffin-embedded, sectioned and stained with hematoxylin and eosin (H&E). Histopathological changes were observed under a light microscope, five visual fields were observed and eosinophil counts were recorded.

### 3.9. Statistical Analysis

The data are shown as the mean ± standard deviation (SD). The statistical analyses were performed using SPSS 16.0.

## 4. Conclusions

In this paper, the composition of the paeonol temperature-sensitive *in situ* gel was screened by thermodynamic and rheology studies, and the optimal composition was determined to be 2% paeonol inclusion, 22% P407, 2% P188 and 2% PEG6000. Nasal toxicity and *in vitro* release were also investigated, and the results of these studies showed that the paeonol thermosensitive gel had low ciliary toxicity and could be used for intranasal administration. The *in vitro* release data were in line with the Higuchi equation, indicating that the paeonol could be absorbed into body through the mucous membranes and have some sustained effect. Finally, the preliminary efficacy of the paeonol thermosensitive *in situ* gel was evaluated with the guinea pig RA model. These results suggested that the paeonol thermosensitive *in situ* gel can significantly reduce IgE and LTE_4_ levels and the number of eosinophils, in addition to improving the pathological changes in the nasal mucosa of the guinea pig. This study provides the basis and a new direction for the treatment of allergic rhinitis.

## Figures and Tables

**Table 1:** Time of cilia continuing swinging of paeonol temperature-sensitive *in situ* gel (*n* = 6).

Items	Physiological saline	Ephedrine hydrochloride	1% Sodium deoxycholate	Blank temperaturesensitive *in situ* gel	Paeonol temperature-sensitive *in situ* gel
Continuing swinging time of cilia (min)	701.60 ± 32.7	694. 43 ± 28.21	33.4 ± 5.7 *	676.20 ± 31.3	662.60 ± 30.1
Relative percentage (%)	100.00%	98.98%	4.76% *	96.38%	94.44%

Note: relative percentage = time of experimental group/time of negative control group × 100;

* data for *p* < 0.01

**Table 2:** Content of leukotriene E4 (LTE4) and immunoglobulin E (IgE) in serum of allergic rhinitis (AR) guinea pigs in each group.

Groups	Number of animals	LTE4 (pg/mL)	IgE (pg/mL)
Blank control group (group A)	10	5.37 ± 2.81	0.71 ± 0.45
AR model group (group B)	10	220.88 ± 133.32 ##	141.38 ± 85.06 ##
Rhinocort nasal spray group (group C)	10	32.65 ± 5.60 #*	18.98 ± 6.93 #*
Low-dose of paeonol temperature-sensitive *in situ* gel group (group D)	10	16.63 ± 4.81 #*	7.72 ± 2.06 #*
Middle-dose of paeonol temperature-sensitive *in situ* gel group (group E)	10	11.70 ± 2.43 #**	3.46 ± 1.78 #**
High-dose of paeonol temperature-sensitive *in situ* gel group (group F)	10	10.37 ± 3.03 #**	3.16 ± 2.54 #**

Note—compare with blank control group:

# data for *p* < 0.05;

## data for *p* < 0.01. Compare with model group:

* data for *p* < 0.05;

** data for *p* < 0.01.

**Table 3:** Number of eosinophil in the nasal mucosa of AR guinea pigs in each group.

Groups	Number of animals	Number of eosinophil in the nasal mucosa
Blank control group (group A)	10	4.18 ± 1.66
AR model group (group B)	10	6.46 ± 2.16 ##
Rhinocort nasal spray group (group C)	10	3.60 ± 0.81 ##**
Low-dose of paeonol temperature-sensitive *in situ* gel group (group D)	10	4.60 ± 2.28 ##*
Middle-dose of paeonol temperature-sensitive *in situ* gel group (group E)	10	3.36 ± 1.83 ##**
High-dose of paeonol temperature-sensitive *in situ* gel group (group F)	10	3.89 ± 1.32 ##**

Note—compare with blank control group:

# data for *p* < 0.05;

## data for *p* < 0.01. Compare with model group:

* data for *p* < 0.05;

** data for *p* < 0.01.

## References

[1] Schenkel E.J. (2006). Effect of desloratadine on the control of morning symptoms in patients with seasonal and perennial allergic rhinitis. Allergy Asthma Proc.

[2] Lu B (2002). New Techniques and New Dosage Forms of Drugs.

[3] Gizurason S. (1993). The relevance of nasal physiology to the design of drug absorption studies. Adv. Drug Deliv. Rev.

[4] Zhang C.X., Zhang W.T., Wang D.K. (2006). The research progress of *in situ* gel, new type of drug delivery system. Chin. J. Hosp. Pharm.

[5] Ning L.L. (2007). Characteristics of ophthalmic *in situ* gel and issues in progress. Chin. J. New Drugs.

[6] Schmolka I.R., Tarcha P.J. (1991). Poloxamers in the pharmaceutical industry. Polymers for Controlled Drug Delivery.

[7] Chou T.C. (2003). Anti-inflammatory and analgesic effects of paeonol in carrageenan evoked thermal hyperalgesia. Br. J. Pharmacol.

[8] Ma Y.L., Bates S.S., Gurney A.M. (2006). The effects of paeonol on the electrophysiological properties of cardiac ventricular myocytes. Eur. J. Pharmacol.

[9] Kim J., Lee H., Lee Y., Oh B.G., Cho C., Kim Y., Shin M., Hong M., Jung SK., Bae H. (2007). Inhibition effects of Moutan Cortex Radicis on secretion of eotaxin in A549 human epithelial cells and eosinophil migration. J. Ethnopharmacol..

[10] Kim S.H., Kim S.A., Park M.K., Kim S.H., Park Y.D., Na H.J., Kim H.M., Shin M.K., Ahn K.S. (2004). Paeonol inhibits anaphylactic reaction by regulating histamine and TNF-a. Int. Immunopharmacol.

[11] Tang H.Y., Yang S., Wang J.B. (2004). Verview of preparation process, formulation reform and clinical application of paeonol. Jiangsu J. Trad. Chin. Med.

[12] Hirlekar R., Kadam V. (2009). Preformulation study of the inclusion complex irbesartan-β-cyelodextrin. AAPS PharmSciTech.

[13] Song T., Wang D.K., Gao H., Zhang C.X., Ji B. (2006). Thermodynamic and rheological properties of therm osensitive gels for nasal delivery of ribavirin. Chin. J. Pharm.

[14] De Santana D.C., Pupo T.T., Sauaia M.G., da Silva R.S., Lopez R.F.V. (2010). Nitric oxide photorelease from hydrogels and from skin containing a nitro-ruthenium complex. Int. J. Pharm.

[15] Jug M., BEeIREvIe-Lacan M., Bengez S. (2009). Novel cyclodextrin-based film form ulation intended for buecal delivery of atenolol. Drug Dev. Ind. Pharm.

[16] Xu X.Y. (1997). The Identification of principal components of clathrate and complex with infrared spectral subtraction method. Chin. J. Pharm. Anal.

[17] Lu W.Y., Chen P.R., Lin G.Q. (2008). New stereoselective synthesis of thiamphenicol and florfenicol from enantiomerically pure cyanohydrin: A chemo-enzymatic approach. Tetrahedron.

[18] Schmolka I.R. (1972). Artificial skin I. Preparation and properties and properties of pluronic F-127 gels for the treatment of burns. J. Biomed. Mater. Res.

[19] Mao S., Shi Z., Bi D. (1997). Research of nasal absorption of dipyrone solution. Chin. Pharm. J.

[20] Song C.J., Wang Y., Wang C.Y. (2008). Preparation and evaluation *in vivo* of scutellaria thermosensitive gel. China J. Chin. Mat. Med.

[21] Miyazaki S., Nakamura T., Takada M. (1991). Thermo-sensitive sol-gel transition of Pluronic F-127. Yakuzaigaku.

[22] Azade T., Fatemeh A., Rassoul D. (2011). Temperature-responsive and biodegradable PVA: PVP k30: poloxamer 407 hydrogel for controlled delivery of human growth hormone (hGH). J. Pediatr. Endocr. Met.

[23] Gratieri T., Gelfuso G.M., Rocha E.M., Sarmento V.H., de Freitas O., Lopez R.F.V. (2010). A poloxamer/chitosan *in situ* forming gel with prolonged retention time for ocular delivery. Eur. J. Pharm. Biopharm.

[24] National Pharmacopoeia Committee (2010). Chinese Pharmacopoeia.

[25] Jiang X.G., Cui J.B., Fang X.L., Wei Y., Xi N.Z. (1995). Toxicity of drugs on nasal mucocilia and the method of its evaluation. Acta Pharm. Sin.

[26] Zhang Q., Jiang X., Xiang W., Lu W., Su L., Shi Z. (2004). Preparation of nimodipineloaded microemulsion for intranasal delivery and evaluation of the targeting efficiency to brain. Int. J. Pharm.

